# A Systematic Review of Continuum Modeling of Skeletal Muscles: Current Trends, Limitations, and Recommendations

**DOI:** 10.1155/2018/7631818

**Published:** 2018-12-06

**Authors:** Tien Tuan Dao, Marie-Christine Ho Ba Tho

**Affiliations:** Sorbonne University, Université de Technologie de Compiègne, CNRS, UMR 7338 Biomechanics and Bioengineering, Centre de Recherche Royallieu, CS 60 319 Compiègne, France

## Abstract

Finite elasticity theory has been commonly used to model skeletal muscle. A very large range of heterogeneous constitutive laws has been proposed. In this review, the most widely used continuum models of skeletal muscles were synthetized and discussed. Trends and limitations of these laws were highlighted to propose new recommendations for future researches. A systematic review process was performed using two reliable search engines as PubMed and ScienceDirect. 40 representative studies (13 passive muscle materials and 27 active muscle materials) were included into this review. Note that exclusion criteria include tendon models, analytical models, 1D geometrical models, supplement papers, and indexed conference papers. Trends of current skeletal muscle modeling relate to 3D accurate muscle representation, parameter identification in passive muscle modeling, and the integration of coupled biophysical phenomena. Parameter identification for active materials, assumed fiber distribution, data assumption, and model validation are current drawbacks. New recommendations deal with the incorporation of multimodal data derived from medical imaging, the integration of more biophysical phenomena, and model reproducibility. Accounting for data uncertainty in skeletal muscle modeling will be also a challenging issue. This review provides, for the first time, a holistic view of current continuum models of skeletal muscles to identify potential gaps of current models according to the physiology of skeletal muscle. This opens new avenues for improving skeletal muscle modeling in the framework of in silico medicine.

## 1. Introduction

Human skeletal muscle is the motor of the locomotion function of the human body. This specific living tissue has complex multiscale and hierarchical architecture (i.e., from fibers to myofibrils, sarcomeres, and contractile proteins (actin and myosin)) and function (e.g., voluntary contraction control) [1–4]. Hierarchical bundles of assembled fibers and fibrils, which are formed by tropocollagen molecules with a helix structure, are basic building constituents of skeletal muscles. The organization of hierarchical fibers and their activation mechanism allow the whole muscle contraction to occur. Moreover, a passive matrix of connective tissues contributes into the force generation process in a cooperative manner within fibers. Skeletal muscle activation mechanism starts by a progressive activation in time and in space of multiple motor units (MU) due to a neural command generated through motor neuron axons from the nervous system. Recruited MU's number, size, morphology, and their behaviors (e.g., firing rate or patterns) determine the activation level and produced mechanical force [5]. Note also that the action potential transmission allows the voltage-sensitive protein (i.e., sarco(endo) plasmic reticulum ATPases SERCA2a in the sarcoplasmic reticulum) to change its shape to open calcium release channel. Then, calcium ions bind to troponin to change its shape allowing tropomyosin to move to the actin side to enable the contraction process at the sarcomere level. Myosin reaches forward, binds to actin, contracts, and releases actin. Then, this protein reaches forward again to bind actin in a new cycle. This interactive and cycling process allows muscle mechanical force to be produced [1, 6, 7]. Skeletal muscle exhibits commonly a nonlinear behavior during dynamic movements. Critical experiments have been done to characterize the skeletal muscle in *in vitro* as well as in *in vivo* conditions [8–11]. However, due to the complex nature of the skeletal muscle, some physical quantities cannot be measured in a noninvasive manner. For example, force distribution and intrinsic tissue stress inside the skeletal muscle under isotropic and anisotropic contractions are among the current immeasurable quantities in *in vivo* conditions. Mathematical modeling of the skeletal muscle is the current engineering solution to estimate these quantities [12–14].

One of the landmark mathematical models of the skeletal muscle activation, contraction, and force has been proposed from the well-known experiment performed by Hill in 1938 to elucidate the muscle work and contraction velocity phenomena. Based on this original finding, 1D lumped-parameter model of muscle contraction and force has been developed [15] and this model has been widely used in rigid body musculoskeletal modeling [14]. Despite its compact formulation with only 5 parameters and computational advantage, this 1D lumped-parameter model could not describe complex structural and functional relationships of the skeletal muscle in an accurate manner, especially in the case of muscle diseases (e.g., dystrophy or spasticity) [16–20]. To investigate the skeletal muscle in its complex nature, continuum mechanics approach has been used. The skeletal muscle has usually been modeled as an inhomogeneous and nearly incompressible body. A range of constitutive laws from the simple elastic material to the complex multiscale chemo-electro-mechanical material has been proposed [21–23]. However, these continuum models are very heterogeneous and it is difficult to elucidate the common modeling aspects and to identify potential gaps according to the real physiology of the skeletal muscle. There is a lack of systematic review of these continuum models. This information may allow the right choice of a model for a specific case study. Moreover, model parameters cover a very large range of values. Thus, the determination of currently used ranges of values if available is necessary for the modeling and simulation of the skeletal muscle in the future.

The objective of the present review study was to synthetize and discuss the widely used continuum models of skeletal muscles in the literature. Useful information related to the developed model formulation and parameters were also reported. Moreover, trends and limitations of these models were highlighted to propose new challenging recommendations for future researches.

## 2. Continuum Models of Skeletal Muscles

### 2.1. Review Method

**Figure 1:**
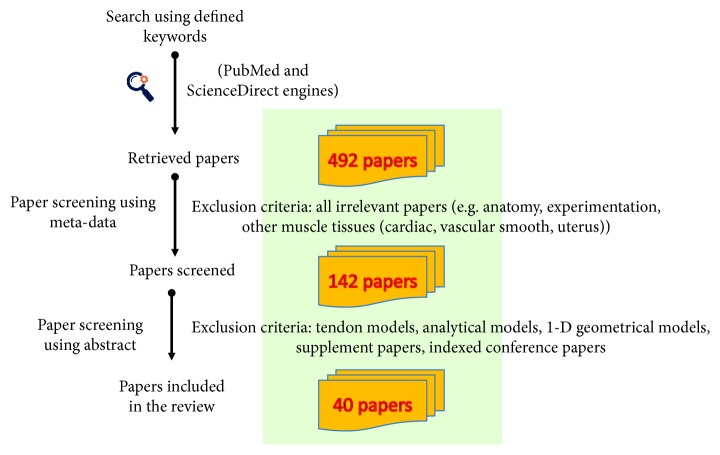
Flowchart of the review process of continuum modeling of the skeletal muscle.

A systematic review process was applied for this present study using two reliable search engines for biomedical literature as PubMed and ScienceDirect. The most widely used continuum models of skeletal muscles were identified and retrieved. Specific keywords (*finite element muscle modeling*, *hyperelastic muscle model, transversely isotropic muscle model, fiber-reinforced muscle model, and muscle stress analysis*) were used. The flowchart of the applied review process is shown in Figure 1. First, meta-data (e.g., title, source) of each paper were initially screened to identify the retrieved papers. All irrelevant papers (e.g., anatomy, experimentation, other muscle tissues (cardiac, vascular smooth, and uterus)) were excluded. The number of screened papers is 142 from 492 retrieved papers. Then, an experienced biomechanical expert of the musculoskeletal system modeling scanned all retrieved results using abstract information to select the most relevant studies. Finally, 40 representative studies (13 passive muscle materials and 27 active muscle materials) were included in this review. Note that exclusion criteria include tendon models, analytical models, 1D geometrical models, supplement papers, and indexed conference papers. Thus, the eligibility criterion focuses on muscle model development and implementation with 3D geometries. Constitutive models with complex muscle network were also included in the review. Note that the search period was set up from 1998 to 2017.

A common structure (i.e., related reference with first author name and the year of publication, muscles, geometries, constitutive laws, simulations, and validation) was used to summarize all retrieved papers. This common structure allows integrating and aggregating the information about the analyzed continuum models: what are the research group and year of work, what are the muscles modeled, how to obtain the geometries, what are the constitutive laws to be used or implemented, and what are the performed simulations and the validation process (if exists) of the developed skeletal muscle models. All analyzed papers were classified into two categories: skeletal muscle as a passive material and skeletal muscle as an active material. A summary of all used and developed continuum models of skeletal muscles was provided to establish common aspects as well as to identify the gaps according to the real physiology of skeletal muscles. Then, respective trends and weaknesses of these models were analyzed and presented. Finally, new challenging recommendations were provided for future researches.

### 2.2. Skeletal Muscle as a Passive Material

Mathematical formulation of skeletal muscle physiology is a complex engineering task. In particular, the consideration of all physiological aspects is practically difficult. One of the most difficult tasks relates to the integration of active behavior of skeletal muscles. However, this leads to complex model formulation and important computational cost. Hence, the modeling of only passive behavior (i.e., consideration of passive matrix of connective tissues and assumption of sarcomere length change in a passive manner) of the skeletal muscle is an acceptable solution under some specific conditions (e.g., virtual surgery simulation where skeletal muscles exhibit commonly a passive behavior or to simulate *in vitro* testing of muscle passive behavior). Classical material laws have been used for modeling the passive behavior of skeletal muscles (Tables 1 and 2). The simplest constitutive behavior is the linear elastic law, which is used to model facial muscles in the nonactive state (e.g., orbicularis oris, zygomaticus major and minor, buccinator and risorius, or depressor anguli oris) and simulate the maxillofacial surgery [22]. This model assumes that the skeletal muscle exhibits as an elastic solid under external mechanical stimuli. It is likely to point out that only fiber fascicles in the passive state and related matrix of connective tissues have been considered in this model formulation. However, it is important to note that this model is anisotropic including also the strain-energy formulation to describe the active state of the skeletal muscles (please refer to Section 2.3 to see the description of this active component). Facial muscles have been modeled in a more complex manner using a fiber-based and orthogonal direction-based elastic material [24] or a nonlinear elastic-viscoplastic model [21] or an orthogonal elastic material [25] or a hyperelastic material using the Mooney-Rivlin formulation [26]. Moreover, skeletal muscles in the upper limbs (subscapularis, supra, and infraspinatus), spine, and lower limbs (ischios, quadriceps, gracilis, sartorius, gastrocnemius, and biceps femoris) have been commonly modeled using a hyperelastic material [27] (based on the Neo-Hookean formulation [28–30] or based on the Mooney-Rivlin formulation [26, 31, 32]) or a nonlinear viscoelastic material [33] or a visco-poroelastic material [34]. In fact, the use of hyperelastic models assumes that the skeletal muscle exhibits a large deformation (>5%) behavior under external solicitation while visco-poroelastic law allows the fluid-filled fiber fascicles and connective tissue to be taken into consideration in model formulation. Finally, orthogonal elastic material allows the fiber orientation to be defined in two different directions with two different constitutive parameters. Only two parameters are needed for the simplest linear elastic law or hyperelastic law based on Neo-Hookean formulation. The use of hyperelastic law based on the Mooney-Rivlin formulation requires three parameters. Note that the number of model parameters increases when more biophysical phenomena are included into constitutive laws. For example, three main parameters are required to formulate the visco-poroelastic material [34]. Six parameters are needed to define the nonlinear elastic-viscoplastic model [21]. Common outcomes of passive muscle materials are muscle stress and strain.

The respective constitutive equations of commonly used hyperelastic material based on the Neo-Hookean and Mooney-Rivlin formulations are expressed as follows:

(1) $$\begin{array}{c} Neo‐Hookean:\;U=C_{10}\left(I_{1}-3\right)+\frac{1}{D}{\left(J-1\right)}^{2}, \end{array}$$

(2) $$\begin{array}{c} Mooney‐Rivlin:\;U=C_{10}\left(I_{1}-3\right)+C_{01}\left(I_{2}-3\right)+\frac{1}{D}{\left(J-1\right)}^{2}, \end{array}$$

where *U* is the strain energy density function; *I*_1_&*I*_2_ are the first and second invariants of the right Cauchy-Green deformation tensor; *C*_10_&*C*_01_&*D* are material constants and their respective used values cover a large range (Table 1); *J* = det(*F*) is the gradient deformation tensor.

Medical imaging techniques (e.g., computed tomography (CT) and magnetic resonance imaging (MRI)) are common data acquisition modalities used to develop 2D and 3D geometrical models of skeletal muscles in *in vitro* and *in vivo* conditions [21, 22, 24–32], except for one study using 3D ideal geometry [34]. There is no clear definition of fiber distribution in passive muscle modeling. The modeling of the skeletal muscle as a passive material has been done in a large range of simulations such as impact simulation [33], maxillofacial surgery [22, 24, 25], facial expressions [26], uniaxial and multiple-axial loadings [28, 29, 31, 32, 34], dynamic movements [27], and aging process [21]. Parameter identification using inverse approach and experimental data was performed using 2D continuum models [28, 29]. Model validation has been commonly performed using experimental data [21, 22, 25, 26, 28, 29, 31, 32, 34] (e.g., postsurgery data for skin envelop [22] or skin deformation from the structured-light scanner [26]). Literature data was also used for comparing with model outcomes [30]. Note that model validation was not performed in two studies [24, 27].

It is noted that the use of passive material may be an acceptable solution for the simulation of virtual surgery procedures in which simulation time is an important factor. Thus, real-time feedback related to skeletal muscle strain and stress during surgical operation may be achieved to provide quantitative indicators for making surgical decisions. Furthermore, when *in vitro* mechanical testing is designed and performed, passive muscle material may be used for model calibration and parameter identification. Generally speaking, most of the passive constitutive laws are appropriate for the designed purposes and available data. However, two studies suffered from the lack of validation making it impossible to evaluate the modeling accuracy and outcome precision [24, 27].

### 2.3. Skeletal Muscle as an Active Material

The modeling of the skeletal muscle as an active material requires the integration of hierarchical fibers and their activation mechanism. Different constitutive laws have been proposed and developed for modeling active skeletal muscles (Tables 3–6). Transversely isotropic behavior has been commonly described in most of developed constitutive models. A specific case of orthotropic behavior has been also proposed [41, 42]. Most of the developed laws have been inspired from the Hill-type phenomenological model including passive and active components of the skeletal muscle. It is important to emphasize that the hyperelastic behavior has been included in most of developed models to describe the skeletal muscle passive component [48, 52]. The active component is commonly defined by the relationship between the fiber activation, stretch, and force components. Note that these force components include commonly the passive and active fiber forces.

#### 2.3.1. Modeling Strategies

Two modeling strategies have been commonly adopted. The first one focuses on the modeling of only the mechanical aspect of skeletal muscles [22, 26, 36–39, 41–48, 53–59] while the second approach performs the coupling between electrical and mechanical aspects to develop an electromechanical model [21, 49, 50]. Mechanical formulation has taken only the force-length relationship into consideration. Active and passive muscle states have been mathematically formulated using exponential and quadratic functions. The integration of electrical aspect into mechanical formulation requires the mathematical formulation of action potential generation through ion channels at cell membrane level. The integration of some chemical components has been also done [51]. The number of parameters is significantly important in active skeletal muscle models. Note also that the number of model parameters rapidly increases when more biophysical phenomena are included into constitutive laws. The number of parameters ranges from 5 to 20 parameters [48, 51, 59]. The values of these model parameters and material constants are commonly set up in an empirical manner. Data assumption is always performed, especially in the case of human muscle modeling. One study attempted to measure *in vivo* data related to contraction amplitude to reduce uncertainty in parameter space and then used it to more accurately reproduce the physical behavior of muscle contraction [59]. Parameter identification was also performed using literature data for the level of stretch-induced fascicle activation [52]. Note that common outcomes of active muscle materials are muscle stress/stretch and strains at fiber and whole muscle levels. Other outcomes include muscle activation level and force-velocity relationship. Membrane potential is also estimated with electromechanical models.

#### 2.3.2. Muscle and Fiber Architecture

Despite the complexity in model formulation and evaluation, active muscle modeling has been commonly performed for a large range of muscles including generic muscle tissue [36, 37, 43, 54], brachialis [35], rectus femoris [38, 46], levator ani [40], biceps brachii [23, 39, 55], gastrocnemius [41, 42, 53], tibialis anterior [44, 47, 49–51], biceps femoris longhead [45], soleus [46, 53], ventral interior lateral muscle [48], lumbar spine muscles [52], and facial muscles [22, 26, 56–59]. Geometrical models of skeletal muscles have been reconstructed from medical imaging (CT and MRI) [22, 23, 26, 38, 45, 50, 53–59]. However, some studies also used ideal 3D geometries to represent the skeletal muscles [36, 37, 43, 44, 47–49, 51, 54].

The definition of fiber architecture is a particular characteristic of the active muscle modeling. Several approaches have been proposed. Parallel fiber distribution in a single direction [22, 37, 39, 41, 42, 44, 46, 48, 51, 54, 55, 59] or at a specific pennation angle [23, 43, 49] has been commonly performed. Bipennate fiber orientation has been also proposed [38]. The definition of fiber according to loading direction has been performed [47]. Fusiform fiber distribution has been established in some models [23, 50]. In particular, mapping technique from different fiber templates showed the important effect of fiber definition in model outcomes [45]. Other approaches like circularly directed and transversely oriented fibers [36] or fiber tangent interpolation using B-spline [56] or curvature-driven cable elements [58] or fiber angle interpolation using piecewise linear functions [26] have been also proposed. Ultrasound images have been used to measure fascicle orientation [53].

#### 2.3.3. Loading Scenarios

Current simulations of active skeletal muscles relate to basic loading scenarios. Isometric activation has been simulated in some studies [36, 50, 51, 55]. Shortening and lengthening have been studied [35, 39, 43, 45, 47, 48, 51]. Shear and deformation in several planes have been also performed [37, 41, 42, 46]. Standing posture and lying position were also simulated [52]. In particular, some studies have attempted to simulate the contribution of active skeletal muscle in the generation of a dynamic movement like knee flexion [38] or plantar flexion [53] or mastication [57] or orofacial movement [58] or facial mimics and expressions [26, 56, 59]. A simulation of surgical gesture on the face has been also performed [22].

#### 2.3.4. Implementation

The implementation of active skeletal muscle models requires specific programming skills. There are no existing commercial finite element programs or solvers allowing to provide active skeletal muscle material. This material is available in FEBio (Musculoskeletal Research Laboratories (MRL), University of Utah, USA), an open source program [60]. The use of the most widely used finite element programs like Abaqus or ANSYS requires the development of user-defined material subroutines (UMAT) [22, 54, 58, 59]. Complex model formulations may be also implemented with other FE codes like nonlinear NIKED [39] or PAK [41] or CMISS [38]. It is important to note that the information related to the computational time and cost is unavailable in the most of published models.

#### 2.3.5. Validation

Mathematical modeling of biological tissues and systems is meaningful only when the model outcome is quantitatively validated in a systematic way. Most of the developed active skeletal muscle models have been validated against literature data [36, 37, 43, 49, 50, 57] or measured data in *in vivo* conditions [22, 26, 39, 41, 42, 44, 47, 53–55, 58, 59]. However, there are still some proposed models without validation efforts [23, 35, 38, 45, 46, 48, 56]. Data used for validation purpose covers a large range of types such as length-force relationship, stress-strain relationship, velocity profile, and shape deformation. It is important to note that the acquisition of accurate *in vivo* measurements at fascicle and whole muscle levels remains a challenge. In particular, *in vivo* human muscle force and stress may not be measured in a noninvasive manner leading to the limited validation capacity in the current continuum muscle models. Moreover, parameter calibration and identification for active muscle material suffer from the lack of experimental data. Among the developed models, outside shape deformation is usually used as an indirect measurement for validation purpose [22, 26, 41, 42, 59]. This information is commonly acquired from imaging data.

Different mathematical formulations of the active muscle material have been developed and proposed in the literature. Models with mechanical behavior have been developed and calibrated for different loading scenarios. Simulation outcomes are fairly consistent with experimental data. This choice is mostly accepted by the continuum muscle modeling community. Thus, the mechanical model of active muscle material could be used and extended for further investigations related to the musculoskeletal biomechanics of the human body [39, 41, 55]. Electromechanical formation has been recently proposed but significant efforts dealing with model calibration and parameter identification need to be done before their use in real application, especially in clinical applications [23, 49]. Moreover, it is important to emphasize that several studies suffered from the lack of systematic validation for the active behavior of the skeletal muscle models [23, 35, 38, 40, 45, 48]. Thus, it is difficult to evaluate the modeling accuracy and outcome precision according to the specific purposes of these studies.

### 2.4. Summary

Passive skeletal muscle modeling involves the use of classical mechanical materials ranging from the simplest one (e.g., linear elastic) to more complex ones (e.g., hyperelastic). Thus, the modeling effort could be optimized when no active muscle behavior is required. In particular, the use of hyperelastic isotropic material based on the Mooney-Rivlin model is an acceptable approximation of the nonlinear behavior of skeletal muscle while keeping a cheaper computational cost. However, how to set up the accurate and reliable values of model parameters remains a challenging issue, especially in the case of more complex constitutive laws [21, 34, 61].

Regarding active skeletal muscle modeling, phenomenological and biophysical modeling approaches are the most widely used to establish respective constitutive laws. Phenomenological modeling attempted to describe mathematically empirical relationships of biological phenomena inside the skeletal muscle. These relationships are consistent with fundamental theory, but they are not directly derived from theory [62–64]. Measured values are usually required to define these relationships. Biophysical modeling [65, 66] establishes mathematical formalizations of the physical properties of the skeletal muscle system. Note that current active muscle models are classified into two categories: mechanical models and electromechanical models. Mechanical models describe the force distribution, internal tissue loading, and shape deformation of skeletal muscle. Electromechanical models allow the electrophysiological aspects of fibers to be integrated into mechanical formulation. It is important to note that electromechanical formulation is the most relevant muscle model. This coupling allows the integration of action potential propagation behavior from the brain to the muscle fibers to be performed. Thus, novel parameters (e.g., stimulus current, transmembrane potential, and intracellular and extracellular conductivity) have been incorporated into the mechanical model formulation [38, 67]. The reported ranges of values for main mechanical and electromechanical model parameters are depicted in Table 7.

Geometrical representation of the whole muscle and fiber representation have been achievable using medical imaging. Outside muscle shapes are usually regular. Meshed models have been generated directly from medical images with a good accuracy due to the outside shape simplicity of the skeletal muscle [38, 45, 59]. Hence, there is no need for specific meshing refinement and improvement. All 2D or 3D muscle models have been meshed using classical meshing algorithm and process. Regarding the upper and lower limb muscles, most of the developed models have been simulated with a single muscle configuration [38, 39, 51]. Only few studies incorporated multiple muscles into a system level [53, 69]. However, multiple muscle configuration is commonly performed for facial modeling [26, 56]. Model parameter identification has been performed in some 2D studies [28, 29]. Simulations of active and passive skeletal muscle behaviors have been performed in a large range of cases from simple loading (e.g., isometric activation and contraction) to complex loading (e.g., impact simulations, injury mechanisms). Developed muscle models have been carefully validated using literature data related to muscle length-force relationship [37, 51] and experimental data related to muscle length [39], deformation shape [26, 41, 42, 59], stress and strain relationship [44, 54], or stress response [47].

One specific point to note relates to the progressive developments of some research groups to improve the muscle rheological models. An example of such improvement deals with the improvement of loading scenarios and implementation code [41, 42]. Another example is the improvement of model scale from one scale to multiple scale formulations with more complex biophysical phenomena [49, 51, 57]. The simulation of muscle coordination mechanism with more muscles is also an updated outcome from single-muscle simulation [69]. In fact, under the complexity of skeletal muscle physiology, a progressive modeling strategy is a good choice to advance the understanding of the continuum muscle biomechanics.

## 3. Trends and Limitations of Current Continuum Models of Skeletal Muscles

### 3.1. Trends of Current Continuum Models of Skeletal Muscles

Trends of current skeletal muscle modeling relate to 3D accurate representation of the entire skeletal muscle using medical imaging techniques, *in vivo* experiments for parameter identification in passive muscle modeling and model validation, and the integration of several coupled biophysical phenomena into the mathematical formulation.

With the current progresses of biomedical knowledge and information and communication technology (ICT), the use of medical imaging techniques to develop subject/patient specific finite element models of the musculoskeletal system has become a customized approach [70–72]. Medical imaging modalities like MRI and CT scans have been used for accurately reconstructing the 3D entire muscle geometries. Note that an experienced operator with deep anatomical knowledge is required to perform the complex segmentation task for some specific muscles like facial muscles [59]. In fact, image-based muscle modeling has become a customized approach.

Mechanical (e.g., indentation) tests have been commonly performed for parameter identification in passive skeletal muscle models [28, 29]. Most of the developed models have been validated against literature data and experimental measurements ranging from stress-strain relationship to detailed deformation pattern. Note that outside shape deformation could be used as an appropriate metric for comparison between model outcome and measurement.

Mathematical description of skeletal muscle behaviors has been an intensive research interest during the last century. Among the landmark studies, the experimental work performed by Hill [1] allows many phenomenological and biophysical models of skeletal muscles to be developed and tested under different physiological and pathophysiological conditions. Finite elastic theory has been adopted to develop 3D continuum models of skeletal muscles including intrinsic activation mechanism. Hyperelasticity and viscoelasticity have been widely considered and integrated into current models. Thus, viscous and elastic characteristics when undergoing deformation have been described to exhibit time-dependent strain. Single-scale and multiscale models have been also proposed. Note that multiscale muscle models allow understanding of muscle behaviors at macroscopic scale (e.g., shape deformation) while accounting for structural and mechanical properties (e.g., sarcomere length change or fiber stretch) at smaller scales. Electrophysiological aspects of muscle contraction mechanism have been coupled with mechanical components to reproduce skeletal muscle behaviors in a more realistic manner.

### 3.2. Limitations of Current Continuum Models of Skeletal Muscles

Parameter identification for active muscle materials, definition of real fiber distribution, data assumption, and limited simulation case studies are drawbacks of the current skeletal muscle modeling.

Despite the accurate and more realistic representation of the skeletal muscle, active constitutive laws have faced a complex challenge of parameter identification [73–75]. In particular, multiscale and electromechanical materials require a great number of parameters to be calibrated and identified. However, there are no existing active 3D continuum muscle models with fully calibrated and identified parameters.

The activation and contraction behaviors of skeletal muscles depend on their shapes including circular (e.g., orbicularis oris muscle), convergent (e.g., pectoralis major muscle), unipennate (e.g., extensor digitorium muscle), nonfusiform parallel (e.g., sactorius muscle), bipennate (e.g., rectus femoris muscle), parallel-fusiform (e.g., biceps brachii muscle), and multipennate (e.g., deltoid muscle) ones. Moreover, fiber shape patterns link directly to failure behaviors in case of muscle fatigue and rupture. Thus, a realistic fiber architecture needs to be established for each modeled muscle. However, current continuum muscle models suffer from fiber representation simplification or no consideration in passive constitutive laws for fiber architecture definition. A potential mapping approach with different fiber architecture templates has been proposed [39]. However, the ideal characteristic of the template limits the representation according to real and detailed fiber distribution. Moreover, the 3D geometries of spinal muscles are practically difficult to obtain, even if medical imaging data could be used. Due to the deep location and multisegment architecture characteristics, these muscles were modeled with fascicle network modeling [52]. This approach is commonly used in rigid multibody modeling [14]. This is one of the main reasons why muscle modeling in the thoracolumbar region of the spine is underexplored according to the upper and lower limb muscles in which the acquisition of detailed information at fascicle and whole muscle levels is commonly feasible. The same remark is noted for the neck muscles [76–78]. Regarding facial muscles, only detailed information at the whole muscle level is available [26, 59]. Thus, further studies need to be investigated to get more detailed information at the fascicle level for these facial muscles.

In addition, data assumption and data estimation using a heuristic approach have been commonly performed leading to the impossible determination of accuracy level of proposed models. For example, contraction amplitude was assumed due to the impractical decomposition of muscle length change into elastic and contractile parts [59]. Moreover, activation level was empirically defined for each muscle [26]. Furthermore, a large range of constitutive constants and values have been used, for example, the values used in hyperelastic material based on Mooney-Rivlin formulation [26, 31, 32]. In particular, the determination of input values for active muscle constitutive models is completely empiric and assumed [23, 36–38, 41, 46–51, 53–55, 57].

In addition, a limited range of simulation case studies has been performed using current muscle models. Simple loading cases such as isometric activation and contraction or shortening and lengthening processes have been investigated. These simulations focus only on the basic understanding of skeletal muscle behavior in physiological conditions. In fact, the application of skeletal muscle models in real cases studies especially in pathophysiological conditions still remains a challenging objective to be achieved.

## 4. Recommendations for Future Researches

Skeletal muscle composition includes mostly water (around 80%), fat, and collagenous tissues. This complex living tissue has been modeled as an anisotropic, viscoelastic, inhomogeneous, nearly incompressible material with large deformation [35, 39, 41, 44, 57]. Moreover, the integration of fibers and their activation mechanism make the modeling task of skeletal muscle remains an open research challenge from experimental and numerical perspectives. To improve the current 3D continuum models, new recommendations deal with the incorporation of multimodal data derived from medical imaging, the integration of more biophysical phenomena, and model reproducibility. Accounting for data uncertainty in skeletal muscle modeling will be also a challenging issue. It is important to note that these recommendations were done based on the best of our knowledge, some aspects may be already achieved in the literature. Consequently, these recommendations should be considered with updated literature review by using more specific keywords.

### 4.1. Incorporation of Multimodal Data Derived from Medical Imaging for Skeletal Muscle Modeling

Imaging techniques like MRI-based ones (classical, cine phase contrast, dynamic, elastography), CT, ultrasound, or optical microendoscopy should be investigated to provide morphological (e.g., fiber and sarcomere length), mechanical (e.g., shear modulus, viscosity), and functional (e.g., contraction velocity) properties of skeletal muscles for enhancing model formulation and validation [79–83]. Thus, a systematic multiscale characterization of the skeletal muscle to provide a single coherent and consistent data set should to be performed. In particular, diffusion tensor imaging opens a new avenue for tracking and reconstructing the fiber distribution in an *in vivo* and realistic way [84, 85]. Thus, robust data processing protocols (e.g., model registration from multimodal and multiscale data or real-time tracking of muscle fiber distribution and contraction velocity) will be also needed to cope with new multimodal data extraction. All these multiscale and multimodal data will lead to robust model formulation, validation, and parameter identification.

In addition, there is no existing experimental technique to measure muscle deformation, stress, and forces in a noninvasive manner. Hence, new original and innovative techniques and measuring protocols need to be developed to make these measurements possible for enhancing model validation. The measurement of contractile properties of the skeletal muscle in *in vivo* conditions at protein level using high-speed atomic force microscopy (HS-AFM) [86] requires more investigations to elucidate the fundamental activation behavior of skeletal muscles. Finally, the control mechanisms in the spinal cord, peripheral, and central nervous system needs to be characterized to elucidate the neural excitability characteristics and function.

### 4.2. Integration of More Biophysical Phenomena

Despite a large range of physical phenomena that existed in current models, all physiological aspects of skeletal muscles are not fully integrated (e.g., lack of muscle remodeling mechanism). Mathematical formulations of new aspects could be investigated. For example, the release mechanism of inorganic phosphate in the formation process of the dominant force-generating cross-bridge state should be incorporated to describe the mechanochemical events of the energy-transducing mechanism [6]. Moreover, the integration of muscle oxidative capacity [10] will lead to a more reliable simulation of the skeletal muscle in physiological conditions. Furthermore, the consideration of muscle remodeling mechanism will make constitutive laws more realistic for muscle damage simulation and recovery mechanics [87–89]. The coupling between multiscale modeling and additive manufacturing technology should be done to design biomimetic muscle-like material reproducing skeletal muscle behaviors in a more realistic manner. In addition, even if the electromechanical model of the skeletal muscle has already incorporated the action potential generation mechanism, some missing processes like progressive motor unit (MU) recruitment in time and space should be included to describe more accurately the muscle activation behavior [5]. All these perspectives could be implemented using new user-defined material subroutines and parameter identification should be performed with new experimental data.

### 4.3. Model Reproducibility

The modeling of skeletal muscles is a complex engineering task. The reproducibility of developed models will rapidly advance the knowledge and applications of skeletal muscle biomechanics [90, 91]. However, there are no existing open access muscle models. Muscle material has been available in FEBio FE computing code but it requires significant modeling efforts to use. Moreover, only mechanical behavior is taken into consideration in this material. Consequently, a common model development guideline needs to be established and developed models should be publically available in open repositories for the muscle modeling community to test and reuse them. In particular, the use of commercial FE code like Abaqus requires the development of complex user-defined material subroutines (e.g., UMAT or VUMAT). There are several research groups developing their models by using this approach [35, 52, 54, 59]. Thus, a future action to share the developed subroutines may advance rapidly the efforts done by each group. Then, the continuum muscle modeling community could benefit from this sharing strategy to achieve a high level of accuracy and physiological meaning of the skeletal muscle models.

### 4.4. Uncertainty Quantification in Skeletal Muscle Modeling

It is well known that the more complex constitutive laws lead to an increasing number of parameters. Due to the use of data assumption (e.g., empiric definition of muscle activation level [26] or simplification of muscle contraction amplitude [59]), data uncertainties should be taken into consideration in numerical muscle modeling and simulation. Thus, probabilistic muscle modeling and simulation should be performed to provide more reliable outcomes [92, 93]. In particular, random uncertainty due to the variability of muscle intrinsic properties and human errors (intersubject, intrasubject, interoperator, and intraoperator) should be modeled. Moreover, epistemic uncertainty due to the modeling hypothesis and limited experiments needs to be accounted for estimating the confidence level of the simulation outcome under a specific modeling purpose.

## 5. Conclusions

Skeletal muscle modeling plays an important role in the understanding of locomotion function of the human body in physiological and pathophysiological conditions. The choice of an appropriate material to model skeletal muscle under a specific condition remains a challenging issue. This review provides, for the first time, a holistic view of current continuum models of skeletal muscles to identify potential gaps of these models according to the real physiology of the skeletal muscle. This opens new avenues for improving skeletal muscle modeling in the framework of in silico medicine.

## Figures and Tables

**Table 1:** Constitutive laws for passive muscle modeling (I).

References	Muscles	Geometries	Constitutive laws	Simulation	Validation
Beldie et al. [22]	20 facial muscles	1 patient, 3D geometries from MRI data	Linear elastic material (*E* = 6.2 kPa, *v* = 0.49)	Maxillofacial surgery	*In vivo* postsurgery data (skin envelop)
Chabanas et al. [24]	6 facial muscles	6 patients, muscles modeled as embedded group elements	Fiber-based and orthogonal direction-based elastic material (*E* = 6.2 kPa and E-fiber = 110 kPa)	Bone repositioning	No
Büchler et al. [27]	Subscapularis, supra, and infraspinatus	Two human fresh frozen cadavers, 3D geometries from CT images	Hyperelastic, and incompressible material *α* = 0.12 MPa, *β* = 1.0	Internal and external rotations of the shoulders	No
Hedenstierna and Halldin [33]	Neck muscles	1 healthy subject, 3D geometries from MRI data	Ogden hyperelastic, viscoelastic material (LS-DYNA FE code) *μ*_*i*_ = 13337&*α*_*i*_ = 14.5	Impact simulations	Resulting head and vertebral kinematics
Barbarino et al. [21]	20 facial muscles	1 healthy subject, 3D geometries from MRI data	Nonlinear elastic-viscoplastic model (6 parameters) (UMAT Abaqus)	Aging	*In vivo* MRI-based displacement
Avril et al. [28]	Calf muscles	1 healthy subject, 2D geometries from MRI data	Hyperelastic material (Neo-Hookean model) *C*_10_ = [9.4–12.9] kPa *K* = [61–78] kPa	Compression garments	*In vivo* MRI measurement (deformed geometries)
Kim et al. [25]	Facial muscles	4 patients, 3D geometries from atlas	Orthogonal elastic material (E_acrossFiber = 0.79 MPa, E_alongFiber = 0.5 Mpa, *v* = 0.43)	Cranio-maxillofacial (CMF) surgery	Postoperative CT scan data (distance map)

**Table 2:** Constitutive laws for passive muscle modeling (II).

References	Muscles	Geometries	Constitutive laws	Simulation	Validation
Wang et al. [31]	Calf muscles	1 healthy subject, 2D geometries from MRI data	Hyperelastic material (Mooney-Rivlin model) *C*_10_ = 1310 Pa, *C*_01_ = −961 Pa, *C*_11_ = 886 Pa	Outside compression	*In vivo* MRI measurement (deformed geometries, cross-sectional area reduction)
Wu et al. [26]	20 facial muscles	1 healthy subject, 3D geometries from MRI data	Hyperelastic material (Mooney-Rivlin model) *C*_10_ = 2.5 kPa, *C*_01_ = 1.175 kPa	Facial expressions	Skin deformation from the structured-light scanner
Affagard et al. [29]	Ischios, quadriceps, gracilis, and sartorius	1 healthy subject, 2D geometries from MRI data	Hyperelastic material (Neo-Hookean model) *C*_10_ = [1.75–3.75] kPa, *D* = 18 MPa^−1^	Contention, compression, and indentation	Ultrasound displacement measurement
Zöllner et al. [30]	Gastrocnemius	1 healthy subject, 3D geometries from MRI data	Hyperelastic material (Neo-Hookean model) *λ* = 0.714 N/mm^2^ and *μ* (*G*) = 0.179 N/mm^2^	High heel posture	Qualitative comparison with literature
Lee et al. [32]	Generic (back spine muscles)	3 healthy subjects, 3D geometries from scanning	Hyperelastic material (Mooney-Rivlin model) *C*_10_ = 1.65 kPa, *C*_01_ = 3.35 kPa	Contact pressure simulation	Contact pressure measurements
Wheatley et al. [34]	Biceps femoris	Ideal 3D cuboid form geometries	Visco-poroelastic material (FEBio) *k*_0_ = [4.250](*m*^4^/*N* − *s*); *M* = [0.16 10]; *α* = [0.12]	Compression	*In vitro* permeability measurement

**Table 3:** Constitutive laws for active muscle modeling (I).

References	Muscles	Geometries	Fiber architecture	Constitutive laws	Simulation	Validation
Martins et al. [35]	Brachialis	3D geometry from imaging data of the Visible Human Project	Fibers aligned in one direction	Active transversely isotropic, hyperelastic, and quasi-incompressible material (5 parameters, Abaqus UMAT routine)	Elongation	No
Johansson et al. [36]	Generic	Ideal geometries	Circularly directed and transversely orientated fibers	Active fiber-driven hyperelastic material	Isometric activation, isokinetic shortening, and stop quick release	Comparison with literature
Yucesoy et al. [37]	Generic	Ideal 3D geometries	Parallel fiber distribution	Active linked fiber-matrix mesh material	Activation, shear	Comparison with literature (muscle length-force characteristics)
Fernandez et al. [38]	Rectus femoris	1 patient, 3D geometries from MRI data	Bipennate fiber orientation	Active orthotropic material	Flexion	No
Blemker et al. [39]	Biceps brachii	Ideal geometries	Parallel fascicles with equal and unequal lengths, curved fascicles	Active fiber-reinforced composite with transversely isotropic material	Shortening	Measured length
d'Aulignac et al. [40]	Levator ani muscle	72-year-old female cadaver, 3D geometries from MRI data	Fibers aligned in one direction	Active transversely isotropic, hyperelastic, and quasi-incompressible material (5 parameters, Abaqus UMAT routine)	Deformation under pressure and muscle contraction	No
Tang et al. [41, 42]	Gastrocnemius	1 frog, 3D geometries from elliptical cross sections	Parallel fiber distribution	Active orthotropic material	Deformation in several planes	Measured deformation shape

**Table 4:** Constitutive laws for active muscle modeling (II).

References	Muscles	Geometries	Fiber architecture	Constitutive laws	Simulation	Validation
Chi et al. [43]	Generic	Simplified muscle-tendon geometries	Parallel fibers at a pennation angle	Active transversely isotropic hyperelastic material	Shortening	Comparison with literature data
Lu et al. [44]	Tibialis anterior	1 New Zealand white rabbit, ideal 3D geometries	Parallel fiber distribution	Active, quasi-incompressible, transversely isotropic, and visco-hyperelastic composite material (14 parameters)	Elongation	Measured stress and strain
Rehorn and Blemker [45]	Biceps femoris longhead	1 heathy subject, 3D geometries from MRI data	Mapping technique	Active fiber-reinforced composite with transversely isotropic material	Lengthening contractions	No
Sharafi and Blemker [46]	Rectus femoris and soleus	1 rabbit, 3D geometries from histological cross sections	Parallel fiber distribution in a single direction	Active hyperelastic, nearly incompressible, transversely isotropic material	Macroscopic shear	No
Ehret et al. [47]	Tibialis anterior	Ideal geometries	Loading-driven fiber direction	Active transversely isotropic material	Shortening and lengthening	Experimental stress response
Böl et al. [23]	Biceps brachii	1 healthy subject, 3D geometries from MRI data	Fusiform fiber orientation at a pennation angle	Active electromechanical material based on the transversely isotropic law (13 parameters)	Contraction	No
Paetsch et al. [48]	Ventral interior lateral	1 tobacco hornworm caterpillar *Manduca sexta*, ideal geometries	Parallel distribution to the longitudinal direction	Active transversely isotropic nonlinear hyperelastic material (20 parameters)	Uniaxial extension	No

**Table 5:** Constitutive laws for active muscle modeling (III).

References	Muscles	Geometries	Fiber architecture	Constitutive laws	Simulation	Validation
Röhrle et al. [49]	Tibialis anterior	Ideal 3D geometries	Parallel fiber distribution at a pennation angle	Active multiscale electromechanical material	Activation	Qualitative comparison with literature
Hernández-Gascón et al. [50]	Tibial anterior	1 rat, 3D geometries from MRI	Fusiform form	Active electromechanical material (thermodynamically consistent constitutive model) (11 parameters)	Isometric and concentric contractions	Comparison with literature
Heidlauf and Röhrle [51]	Tibialis anterior	Ideal 3D fiber geometries	Parallel fiber distribution along the edge of the cuboid	Active multiscale chemo-electro-mechanical material (20 parameters)	Shortening, isometric contraction	Comparison with literature (muscle length-force characteristics)
Toumanidou and Noailly [52]	Lumbar spine muscles	3D muscle network from anatomical landmarks	Fibers aligned in one direction	Active transversely isotropic, hyperelastic, and quasi-incompressible material (8 parameters, UMAT Abaqus)	Standing posture and lying position	Comparison with literature (intradiscal pressure)
Kinugasa et al. [53]	Gastrocnemius and soleus	1 healthy subject, 3D geometries from MRI data	Fascicle orientation measurement from ultrasound images	Active transversely isotropic, hyperelastic, and quasi-incompressible material	Plantar flexion	Achilles tendon force measurements
Spyrou et al. [54]	Generic	3D periodic unit cells	Fiber direction along the global *z*-axis	Active two-phase composite material (UMAT Abaqus) (14 parameters)	Uniaxial loading	In vitro passive and active stress-strain response measurement (tibialis anterior)
Clemen et al. [55]	Biceps	1 healthy subject, 3D geometries from MRI data	Parallel fiber distribution in a single direction	Active transversely isotropic hyperelastic material	Isometric contraction	Measured indentation data

**Table 6:** Constitutive laws for active muscle modeling (IV): special case of facial muscles.

References	Muscles	Geometries	Fiber architecture	Constitutive laws	Simulation	Validation
Gladilin et al. [56]	20 facial muscles	1 healthy subject, 3D geometries from MRI data	Fiber tangent interpolation using B-spline	Active fibrous material with heuristic model construction	Facial mimics (happiness, disgust)	No
Röhrle and Pullan [57]	Masseter	3D geometries from the Visible Human Project	Parallel fiber distribution using anatomical-based approximation	Active hyperelastic, incompressible, and transversely isotropic material (9 constants)	Mastication	Comparison with literature
Beldie et al. [22]	20 facial muscles	1 patient, 3D geometries from MRI data	Parallel fiber distribution in a single direction	Active, quasi-incompressible, transversely isotropic, and hyperelastic material (13 parameters) (UMAT LS-DYNA)	Maxillofacial surgery	*In vivo* postsurgery data (skin envelop)
Nazari et al. [58]	10 paired facial muscles	1 subject, 3D geometries from CT data	Curvature-driven cable elements	Active transversely isotropic material (ANSYS)	Dynamic orofacial movements	Measured velocity profile and the acoustic signal
Wu et al. [26]	20 facial muscles	1 healthy subject, 3D geometries from MRI data	Fiber angle interpolation by piecewise linear functions	Active heterogeneous force-drivenhyperelastic material	Facial expressions	Skin deformation from the structured-light scanner
Fan et al. [59]	2 paired zygomaticus major	1 healthy subject, 3D geometries from MRI data	Parallel fiber distribution in muscle mean-line direction	Active transversely isotropic, hyperelastic, and quasi-incompressiblematerial (5 parameters) (VUMAT Abaqus)	Facial mimics	*In vivo* MRI-based displacement

**Table 7:** Reported ranges of values for some main mechanical and electromechanical model parameters.

Parameters	Ranges of values	References
Passive Mooney-Rivlin material parameters	*c*_1_ = *c*_2_ = 0.01MPa	Röhrle [68]
Activation level	*α* = [0 → 1]	Blemker et al. [39]; Röhrle and Pullan [57]
Constant Cauchy stress in passive part	*σ*_passive_^ff^ = 0.3MPa	Röhrle and Pullan [57]
Constant Cauchy stress in active part	*σ*_active_^ff^ = 0.3MPa	Röhrle and Pullan [57]
Optimal fiber stretch length	*λ*_ofl_ = 1.4	Blemker et al. [39]; Röhrle and Pullan [57]
Maximal contractile stress	*σ* = 0.03 MPa	Röhrle [68]
Peak stress	*σ*_0_ = [0.46 0.6688]MPa	Martins et al. [35]; Toumanidou and Noailly [52]; Fan et al. [59]
Maximum isometric stress	*σ*_max_ = [0.22 − 0.3]MPa	Tang et al. [41]; Blemker et al. [39]
Resting calcium level	Ca_0_ = 0.01*μ*M	Fernandez et al. [38]
Intracellular calcium concentration	[Ca^2+^]_max_ = 2.5mM	Fernandez et al. [38]
Intracellular conductivity	*σ*_i_ = 0.6mS mm^−1^	Fernandez et al. [38]
Extracellular conductivity	*σ*_e_ = 0.6mS mm^−1^	Fernandez et al. [38]
Surface-to-volume ratio of the cell	*A*_*m*_ = 80mm^−1^	Fernandez et al. [38]
Capacitance of the cell membrane	*C*_m_ = 0.009*μ*F mm^−2^	Fernandez et al. [38]
